# Supplementary material for: Electron Donor and Acceptor Influence on the Nonlinear Optical Response of Diacetylene-Functionalized Organic Materials (DFOMs): Density Functional Theory Calculations
Source: Molecules. 2019 Jun 2;24(11):2096. doi: 10.3390/molecules24112096 (PMC6600272; doi:10.3390/molecules24112096)
Supplement: Supplementary file 1 [file molecules-24-02096-s001.pdf]

Supplementary Materials

Electron Donor and Acceptor Influence on the Nonlinear Optical Response of Diacetylene-Functionalized Organic Materials (DFOMs): Density Functional Theory Calculations

Muhammad Khalid [1,*], Riaz Hussain [2], Ajaz Hussain [3,*] , Bakhat Ali [1], Farrukh Jaleel [1], Muhammad Imran [4], Mohammed Ali Assiri [4], Muhammad Usman Khan [5], Saeed Ahmed [1], Saba Abid [1], Sadia Haq [1], Kaynat Saleem [1], Shumaila Majeed [1] and Chaudhary Jahrukh Tariq [1]

[1] Department of Chemistry, Khwaja Fareed University of Engineering & Information Technology, Rahim Yar Khan 64200, Pakistan; bakhatali@gmail.com (B.A.); Farrukh1002@hotmail.com (F.J.); Jamsaeed41@gmail.com (S.A.); sabaabid206@gmail.com (S.A.); muskanhaq557@gmail.com (S.H.); kainat.saleem221@yahoo.com (K.S.); shumaila.ryk.2016@gmail.com (S.M.); jahrukh@gmail.com (C.J.T.)

[2] Department of Chemistry, University of Education Lahore, D.G. Khan Campus, Dera Ghazi Khan 32200, Pakistan; riaz.hussain@ue.edu.pk

[3] Institute of Chemical Sciences, Bahauddin Zakariya University, Multan 60800, Pakistan

[4] Department of Chemistry, Faculty of Science, King Khalid University, Abha 61413, P.O. Box 9004, Saudi Arabia; imranchemist@gmail.com (M.I.); aliabdullahasirri@gmail.com (M.A.A.)

[5] Department of Applied Chemistry, Government College University, Faisalabad 38000, Pakistan; usman.chemistry@gmail.com

* Correspondence: drajazhussain@bzu.edu.pk or ajazhussain786921@gmail.com (A.H.); khalid@iq.usp.br or muhammad.khalid@kfueit.edu.pk (M.K.)

Table S1. Optimized Cartesian Co-ordinates of Parent compound **R**.

Symbol	X	Y	Z
C	5.439243	-0.856359	0.849802
C	4.052509	-0.858550	0.847818
C	3.322593	-0.004929	-0.005831
C	4.050370	0.850397	-0.859521
C	5.437141	0.854134	-0.859077
C	6.158799	0.000796	-0.003651
H	5.979392	-1.525810	1.514548
H	3.513407	-1.524091	1.514132
H	3.509644	1.516753	-1.523712
H	5.975586	1.520298	-1.528496
C	1.902631	-0.005145	-0.004128
C	0.679977	-0.004852	-0.001220
C	-0.679986	-0.004865	0.001574
C	-1.902638	-0.005005	0.004533
C	-3.322599	-0.004840	0.006123
C	-4.052326	-0.859674	-0.846474
C	-4.050572	0.851630	0.858508
C	-5.439061	-0.857557	-0.848717
H	-3.513051	-1.526053	-1.511809
C	-5.437329	0.855323	0.857793
H	-3.510001	1.518882	1.521929
C	-6.158803	0.000758	0.003384
H	-5.979062	-1.527843	-1.512742
H	-5.975953	1.522231	1.526327
N	7.548238	-0.034397	-0.040551
H	8.000231	0.794379	-0.400152
H	8.001672	-0.393911	0.787472
N	-7.548183	-0.034395	0.040007
H	-8.000425	0.794874	0.398098
H	-8.001511	-0.395736	-0.787270

Table S2. Optimized Cartesian Co-ordinates of Compound **1**.

Symbol	X	Y	Z
C	4.236402	-1.754586	0.112994
C	2.837488	-1.718921	0.113250
C	2.156376	-0.485467	0.032981
C	2.900649	0.700185	-0.044581
C	4.301231	0.661113	-0.047131
C	4.974961	-0.563019	0.033502
H	4.752104	-2.693893	0.172928
H	2.276377	-2.631815	0.170410
H	4.860660	1.574134	-0.110678
C	0.771535	-0.441453	0.023124
C	-0.471625	-0.402004	0.014292
C	-1.902577	-0.359780	0.006620
C	-3.099988	-0.324489	0.000291
C	-4.513761	-0.282700	-0.005115
C	-5.237730	-0.564039	-1.164766
C	-5.194462	0.055045	1.181319
C	-6.646069	-0.504455	-1.151289
H	-4.730706	-0.823321	-2.071129
C	-6.598970	0.124958	1.193472
H	-4.645590	0.262956	2.068832
C	-7.328317	-0.159590	0.023420
H	-7.204143	-0.732129	-2.037690
H	-7.112447	0.389467	2.091122
N	-8.801662	-0.086908	0.039298
H	-9.105814	0.575663	0.721041
H	-9.135821	0.182561	-0.862703
N	6.451476	-0.603682	0.034271
O	7.097622	0.449436	-0.033267
O	7.041893	-1.687530	0.100511
N	2.209011	1.999002	-0.117767
O	2.875012	3.044791	-0.169917
O	0.975816	2.049892	-0.118207

Table S3. Optimized Cartesian Co-ordinates of Compound **2**.

Symbol	X	Y	Z
C	4.893338	0.948856	-0.736010
C	3.493152	0.866075	-0.765760
C	2.836208	-0.126212	-0.034102
C	3.569433	-1.044977	0.720563
C	4.975940	-0.964302	0.752231
C	5.630512	0.040159	0.020295
H	5.402239	1.711155	-1.287565
H	2.926426	1.563782	-1.337160
H	3.060108	-1.792266	1.277319
H	5.545470	-1.662765	1.329465
C	1.513982	-0.156440	-0.025062
C	0.246469	-0.168514	-0.028097
C	-1.127658	-0.181559	-0.031599
C	-2.308166	-0.193584	-0.036205
C	-3.759640	-0.213614	-0.045300
C	-4.503889	0.886608	0.375768
C	-4.405215	-1.373871	-0.512204
C	-5.905501	0.834365	0.301913
H	-4.013996	1.761637	0.745445
C	-5.802788	-1.431340	-0.568043
H	-3.828761	-2.213297	-0.830623
C	-6.549830	-0.315927	-0.179341
H	-6.294751	-2.320767	-0.908255
N	7.101208	0.140100	0.025206
O	7.790721	-0.616640	0.726707
O	7.658737	0.991296	-0.683610
N	-8.018613	-0.349267	-0.267464
H	-8.294823	-1.030131	-0.951596
H	-8.403810	-0.593960	0.625252
N	-6.700929	1.989990	0.728059
H	-6.997991	1.857259	1.674024
H	-6.145530	2.821994	0.664026

Table S4. Optimized Cartesian Co-ordinates of Compound **3**.

Symbol	X	Y	Z
C	-4.476514	-1.717753	-0.401008
C	-3.079975	-1.600822	-0.451582
C	-2.474697	-0.367557	-0.181126
C	-3.249298	0.744462	0.129809
C	-4.652334	0.630279	0.180019
C	-5.259246	-0.607991	-0.086327
H	-4.948807	-2.656801	-0.599361
H	-2.478534	-2.449071	-0.685805
H	-5.256273	1.481115	0.419239
C	-1.142874	-0.295257	-0.174554
C	0.112306	-0.236981	-0.173238
C	1.488306	-0.173221	-0.171943
C	2.671667	-0.118377	-0.172031
C	4.115032	-0.050018	-0.174809
C	4.887059	-0.853905	0.662080
C	4.737268	0.844858	-1.069425
C	6.287122	-0.784764	0.583132
H	4.416393	-1.522250	1.351096
C	6.132892	0.927308	-1.133091
H	4.141720	1.460632	-1.706163
C	6.906234	0.095196	-0.318711
H	6.604352	1.619954	-1.801908
N	-6.725606	-0.753430	-0.051315
O	-7.453284	0.196557	0.276472
O	-7.239831	-1.839327	-0.356924
N	8.374511	0.145136	-0.401473
H	8.644436	0.499023	-1.301421
H	8.731654	0.745820	0.316835
N	7.109280	-1.636249	1.448788
H	7.380048	-1.120221	2.261358
H	6.581592	-2.443170	1.723665
N	-2.596039	2.024853	0.417093
O	-3.283685	2.975866	0.682631
O	-1.394361	2.080660	0.378775

Table S5. Optimized Cartesian Co-ordinates of Compound **4**.

Symbol	X	Y	Z
C	-5.262414	0.786686	0.901942
C	-3.859185	0.755839	0.873603
C	-3.205034	-0.069815	-0.039556
C	-3.923957	-0.881197	-0.911623
C	-5.330306	-0.837934	-0.896310
C	-5.999079	-0.009662	0.018941
H	-5.771201	1.422989	1.599601
H	-5.893082	-1.457113	-1.569368
C	-1.825938	-0.074180	-0.068014
C	-0.579468	-0.044933	-0.058773
C	0.799030	-0.012584	-0.048560
C	1.981668	0.013139	-0.039109
C	3.407423	0.010682	-0.018362
C	4.109114	1.191859	-0.295559
C	4.114954	-1.167652	0.284698
C	5.503002	1.200624	-0.249805
H	3.571235	2.090411	-0.523973
C	5.515047	-1.168995	0.282100
H	3.578242	-2.073336	0.484378
C	6.207399	0.022740	0.041163
N	-7.465169	0.042107	0.035443
O	-8.123714	-0.639370	-0.762717
O	-8.044739	0.762649	0.858415
N	7.676304	0.044874	0.088934
H	7.994227	-0.675442	0.711661
H	8.047801	-0.111199	-0.823051
N	6.227214	2.446232	-0.508556
H	6.463434	2.504827	-1.475905
H	5.649186	3.224221	-0.256089
N	6.255979	-2.411527	0.557252
H	6.921788	-2.574316	-0.169340
H	6.734086	-2.322078	1.433430
H	-3.415428	-1.514400	-1.607561
H	-3.306486	1.359684	1.558452

Table S6. Optimized Cartesian Co-ordinates of Compound **5**.

Symbol	X	Y	Z
C	4.927856	0.612472	-0.263699
C	3.527317	0.616753	-0.272621
C	2.824767	-0.507086	0.178505
C	3.504010	-1.638966	0.613452
C	4.907753	-1.643827	0.627921
C	5.618341	-0.515877	0.193195
H	5.467840	1.473352	-0.598115
H	5.435848	-2.509691	0.975522
C	1.447288	-0.485993	0.194378
C	0.207095	-0.410809	0.177218
C	-1.164080	-0.327690	0.158250
C	-2.357444	-0.255959	0.142550
C	-3.777776	-0.197415	0.124099
C	-4.457095	0.771639	0.864509
C	-4.507798	-1.095259	-0.690260
C	-5.851590	0.836674	0.809950
H	-3.909817	1.451356	1.482498
C	-5.908947	-1.047325	-0.721209
H	-3.994738	-1.848987	-1.251194
C	-6.578120	-0.066827	0.020737
N	7.087166	-0.513489	0.214535
O	7.709692	-1.497201	0.643641
O	7.703675	0.475920	-0.199766
N	-8.041416	0.026702	-0.037149
H	-8.359553	-0.345845	-0.912038
H	-8.440665	-0.495146	0.718266
N	-6.550652	1.866664	1.582763
H	-6.816742	1.497197	2.471491
H	-5.939264	2.648656	1.714597
N	2.807250	1.805795	-0.754469
O	1.579248	1.810542	-0.765343
O	3.445927	2.782701	-1.133062
N	-6.671210	-2.013635	-1.528775
H	-7.310111	-2.508830	-0.950660
H	-7.179931	-1.516531	-2.241001
H	2.951373	-2.492510	0.946687

Table S7. Optimized Cartesian Co-ordinates of Compound **6**.

Symbol	X	Y	Z
C	-4.600216	-1.210491	-0.229092
C	-3.200260	-1.161190	-0.227494
C	-2.546632	0.058077	-0.009084
C	-3.273969	1.224366	0.193663
C	-4.677111	1.176281	0.198659
C	-5.338753	-0.042442	-0.008239
H	-5.102748	-2.141221	-0.389183
H	-5.242094	2.072380	0.364882
C	-1.170990	0.088416	0.020085
C	0.072578	0.063623	0.030180
C	1.446238	0.036231	0.041325
C	2.640299	0.013550	0.052008
C	4.063099	0.004008	0.039705
C	4.767738	-0.773718	0.965349
C	4.769789	0.748519	-0.928908
C	6.164220	-0.791366	0.934334
H	4.237794	-1.337476	1.703319
C	6.172061	0.754149	-0.938901
H	4.236621	1.358879	-1.628674
C	6.867172	-0.034352	-0.014205
N	-6.806556	-0.098576	0.004017
O	-7.471580	0.925182	0.226050
O	-7.379725	-1.173902	-0.210088
N	8.333608	-0.079437	-0.043325
H	8.649539	0.125420	-0.972629
H	8.702510	0.596821	0.596516
N	6.890667	-1.620469	1.899651
H	7.130526	-1.072391	2.699301
H	6.307257	-2.384406	2.180079
N	-2.570951	2.483820	0.414106
O	-3.209649	3.508846	0.605578
O	-1.347768	2.497978	0.410578
N	-2.429423	-2.392957	-0.460980
O	-1.202546	-2.350650	-0.462574
O	-3.025958	-3.449155	-0.641934
N	6.908528	1.569849	-1.918031
H	7.519707	2.195308	-1.445141
H	7.445994	0.962711	-2.513731

Table S8. Natural bond orbital (NBO) analysis of compound **R** using B3LYP/6-31G (d,p).

Donor (i)	Type	Acceptor (j)	Type	E(2)a (kcal/mol)	E(j)_E(i)b (a.u.)	F(i,j)c (a.u.)
C1-C2	π	C3-C4	π^*	14.95	0.29	0.016
C1-C2	π	C5-C6	π^*	21.98	0.28	0.072
C3-C4	π	C1-C2	π^*	22.66	0.28	0.071
C3-C4	π	C5-C6	π^*	17.68	0.27	0.062
C5-C6	π	C1-C2	π^*	15.53	0.29	0.060
C5-C6	π	C3-C4	π^*	25.41	0.28	0.077
C15-C17	π	C16-C18	π^*	22.66	0.28	0.071
C15-C17	π	C20-C22	π^*	17.68	0.27	0.062
C15-C17	π	C15-C17	π^*	0.94	0.27	0.015
C15-C17	π	C16-C18	π^*	22.66	0.28	0.071
C15-C17	π	C20-C22	π^*	17.68	0.27	0.062
C3-C11	∂	C11-C12	∂^*	11.01	1.67	0.121
C5-C10	∂	C4-C5	∂^*	1.10	1.12	0.031
C5-C10	∂	C5-C6	∂^*	0.70	1.08	0.25
C1-C2	∂	C1-H7	∂^*	1.39	1.16	0.036
C1-C2	∂	C2-C3	∂^*	2.76	1.26	0.056
C1-C6	∂	C1-H7	∂^*	1.03	1.16	0.031
C2-C3	∂	C11-C12	π^*	1.60	0.82	0.033
C11-C12	π	C3-C4	∂^*	3.70	0.83	0.050
C15-16	∂	C13-C14	π^*	1.61	0.82	0.033
C11-C12	π	C2-C3	∂^*	1.00	0.42	0.072
N25	LP(1)	C5-C6	π^*	28.53	0.32	0.091
N28	LP(1)	C20-C22	π^*	28.55	0.32	0.091

Table S9. Natural bond orbital (NBO) analysis of **1** using B3LYP/6-31G (d, p).

Donor (i)	Type	Acceptor (j)	Type	E(2)a (kcal/mol)	E(j)_E(i)b (a.u.)	F(i,j)c (a.u.)
C1- C2	π	C3-C4	π^*	21.54	0.28	0.071
C1-C2	π	C5-C6	π^*	19.39	0.27	0.065
C2-C3	σ	C3-C11	σ^*	5.32	1.40	0.077
C3-C4	π	C1-C2	π^*	17.63	0.28	0.065
C3-C4	π	C5-C6	π^*	22.80	0.27	0.070
C3-C11	σ	C11-C12	σ^*	12.34	1.63	0.127
C5-C6	π	C1-C2	π^*	21.08	0.29	0.071
C5-C6	π	C3-C4	π^*	16.29	0.29	0.062
C5-C6	π	N24- O25	π^*	25.34	0.15	0.059
C11-C12	σ	C3-C11	σ^*	12.04	1.51	0.121
C11-C12	σ	C12-C13	σ^*	7.59	1.46	0.094
C11-C12	π	C13-C14	π^*	10.31	0.43	0.059
C11-C12	π	C13-C14	π^*	36.07	0.42	0.046
C11-C12	π	C3-C4	π^*	219.88	0.29	0.072
C 11-C12	π	C13-C 14	π^*	5.72	0.42	0.045
C11-C12	π	C13-C 14	π^*	9.68	0.42	0.058
C12-C13	σ	C11-C 12	σ^*	9.53	11.61	0.111
C12-C13	σ	C13-C14	σ^*	16.82	1.82	0.156
C13-C14	σ	C12-C13	σ^*	12.79	1.51	0.124
C13-C14	σ	C14-C15	σ^*	8.31	1.38	0.096

Table S9. *Cont.*

C13 C14	π	C11-C12	π^*	10.23	0.40	0.057
C13-C14	π	C11-C12	π^*	6.20	0.39	0.045
C13-C14	π	C11-C12	π^*	6.31	0.39	0.045
C13-C14	π	C11-C12	π^*	10.85	0.39	0.059
C13-C14	π	C15- C16	π^*	8.36	0.32	0.050
C14-C15	σ	C13-C14	σ^*	11.84	1.74	0.128
C15-C16	π	C13-C14	π^*	13.24	0.40	0.069
C15-C16	π	C17-C20	π^*	19.49	0.28	0.066
C15-C16	π	C18-C22	π^*	19.11	0.29	0.067
C17-C20	π	C15-C16	π^*	18.82	0.28	0.067
C17-C20	π	C18-C22	π^*	20.66	0.28	0.070
C18-C22	π	C15-C16	π^*	20.79	0.28	0.069
C18-C22	π	C17-C20	π^*	19.42	0.28	0.067
N24-O25	π	N24-O25	π^*	7.72	0.31	0.053
O25	LP	C6-N24	σ^*	13.22	0.56	0.077
O25	LP	N24-O26	σ^*	17.90	0.69	0.100
O26	LP	C6- N24	σ^*	113.05	0.56	0.076
O26	LP	N24-O25	σ^*	17.82	0.69	0.100
O26	LP	N24-O25	π^*	160.05	0.14	0.135
N27	LP	C18-C22	π^*	5.93	0.33	0.043
N27	LP	C20-C22	σ^*	5.08	0.87	0.060
N30	LP	C16-C18	σ^*	5.37	0.86	0.061
N30	LP	C18-C22	π^*	5.68	0.33	0.042
C3-C4	π	C11-C12	π^*	39.41	0.08	0.096
C5-C6	π	C1-C2	π^*	262.40	0.01	0.080
C5-C6	π	C3-C4	π^*	230.12	0.01	0.083
C11-C12	π	C13-C14	π^*	18.24	0.04	0.083
C11-C12	π	C13-C14	π^*	14.11	0.04	0.064
C11-C12	π	C13-C14	π^*	14.50	0.05	0.065
C11-C12	π	C13-C14	π^*	29.14	0.04	0.084
C15-C16	π	C13-C14	π^*	16.83	0.12	0.076
N24-O25	π	C5-C6	π^*	19.94	0.13	0.063

Table S10. Natural bond orbital (NBO) analysis of **2** by using B3LYP/6-31G (d, p).

Donor (i)	Type	Acceptor (j)	Type	E(2)a (kcal/mol)	E(j)_E(i)b (a.u.)	F(i,j)c (a.u.)
C1-C2	∂	C1-C6	∂^*	2.59	1.25	0.051
C1-C2	∂	C1-H7	∂^*	1.26	1.20	0.035
C1-C2	∂	C2-C3	∂^*	2.46	1.26	0.050
C1-C2	∂	C3-C9	∂^*	3.26	1.30	0.058
C1-C2	∂	C6-N21	∂^*	4.51	0.99	0.061
C_1-C_2	π	C_3-C_4	π^*	22.08	0.29	0.072
C_1-C_2	π	C_5-C_6	π^*	18.99	0.27	0.065
C1-C6	∂	C1-C2	∂^*	2.06	1.27	0.046
C1-C6	∂	C5-C6	∂^*	3.89	1.26	0.063
C1-C6	∂	N21-O22	∂^*	2.32	1.14	0.046
C1-H7	∂	C2-C3	∂^*	3.52	1.10	0.056
C1-H7	∂	C5-C6	∂^*	4.26	1.08	0.060
C1-H7	∂	C6-N21	∂^*	0.60	0.83	0.020

Table S10. *Cont.*

C_3-C_4	π	C_1-C_2	π*	17.77	0.28	0.065
C_3-C_4	π	C_5-C_6	π*	22.92	0.27	0.071
C_3-C_4	π	C_9-C_{10}	π*	17.99	0.38	0.079
C_3-C_9	σ	C_9-C_{10}	σ*	11.16	1.64	0.121
C_5-C_6	π	C_1-C_2	π*	20.86	0.29	0.071
C_5-C_6	π	C_3-C_4	π*	16.34	0.29	0.062
C_5-C_6	π	N_{21}-O_{23}	π*	28.49	0.15	0.063
C_9-C_{10}	σ	C_3-C_9	σ*	9.83	1.43	0.106
C_9-C_{10}	σ	C_{10}-C_{11}	σ*	8.42	1.46	0.099
C_9-C_{10}	π	C_{11}-C_{12}	π*	7.94	0.43	0.052
C_9-C_{10}	π	C_{11}-C_{12}	π*	7.62	0.43	0.052
C_9-C_{10}	π	C_3-C_4	π*	15.44	0.30	0.064
C_9-C_{10}	π	C_{11}-C_{12}	π*	7.38	0.43	0.051
C_9-C_{10}	π	C_{11}-C_{12}	π*	7.70	0.43	0.051
C_{10}-H_{11}	σ	C_9-C_{10}	σ*	10.74	1.66	0.119
C_{10}-C_{11}	σ	C_{11}-C_{12}	σ*	16.01	1.81	0.152
C_{11}-C_{12}	σ	C_{10}-C_{11}	σ*	12.02	1.50	0.120
C_{11}-C_{12}	σ	C_{12}-C_{13}	σ	9.50	1.42	0.104
C_{11}-H_{12}	π	C_9-C_{10}	π*	8.19	0.40	0.051
C_{11}-H_{12}	π	C_9-C_{10}	3	8.06	0.40	0.051
C_{11}-C_{12}	3	C_9-C_{10}	π*	8.13	0.40	0.051
C_{11}-C_{12}	3	C_9-C_{10}	3	8.56	0.40	0.052
C_{11}-C_{12}	3	C_{13}-C_{14}	π*	9.68	0.32	0.054
C_{12}-C_{13}	σ	C_{11}-C_{12}	σ*	12.94	1.75	0.135
C_{13}-C_{14}	π	C_{11}-C_{12}	3	15.59	0.40	0.074
C_{13}-C_{14}	π	C_{15}-C_{18}	π*	19.43	0.28	0.066
C_{13}-C_{14}	π	C_{16}-C_{20}	π*	20.45	0.28	0.069
C_{15}-C_{18}	π	C_{13}-C_{14}	π*	19.37	0.28	0.067
C_{15}-C_{18}	π	C_{16}-C_{20}	π*	20.99	0.28	0.070
C_{16}-C_{20}	π	C_{13}-C_{14}	π*	21.04	0.28	0.069
C_{16}-C_{20}	π	C_{15}-C_{18}	π*	20.73	0.28	0.069
O22	LP(2)	C_6-N_{21}	σ*	13.04	0.57	0.077
O22	LP(2)	N_{21}-O_{23}	σ*	18.04	0.69	0.101
N27	LP(1)	C13-C14	σ*	0.54	0.86	0.019
LP-O_{22}	LP(2)	N_{21}-O_{23}	π*	160.28	0.14	0.135
LP-O_{23}	LP(2)	C_6-C_{21}	σ*	12.97	0.57	0.077
LP-O_{23}	LP(2)	N_{21}-O_{22}	σ*	18.10	0.69	0.101
LP-N_{24}	LP(1)	C_{18}-C_{20}	σ*	5.95	0.87	0.065
LP-N_{27}	LP(1)	C_{14}-C_{16}	σ*	5.63	0.87	0.063
LP-N_{30}	LP(1)	C_{18}-C_{20}	σ*	8.01	0.88	0.075
C_3-C_4	π	C_9-C_{10}	π*	27.82	0.09	0.088
C_5-C_6	π	C_3-C_4	π*	224.17	0.01	0.083
C_9-C_{10}	π	C_{11}-C_{12}	π*	20.40	0.04	0.081
C_9-C_{10}	π	C_{11}-C_{12}	π*	18.10	0.04	0.081
C_{13}-C_{14}	π	C_{11}-C_{12}	π*	19.18	0.12	0.081
N_{21}-O_{23}	π	C_5-C_6	π*	18.10	0.13	0.061

Table S11. Natural bond orbital (NBO) analysis of **3** using B3LYP/6-31G (d,p).

Donor (i)	Type	Acceptor (j)	Type	E(2)a (kcal/mol)	E(j)_E(i)b (a.u.)	F(i,j)c (a.u.)
C1-C2	∂	C1-C6	∂*	2.53	1.26	0.061
C1-C2	∂	C1-C6	∂*	1.32	1.21	0.036
C1-C2	∂	C2-C3	∂*	2.16	1.25	0.045
C1-C2	∂	C3-C10	∂*	3.04	1.38	0.058
C1-C2	∂	C6-C23	∂*	4.47	0.99	0.061
C1-C2	π	C3-C4	π*	23.57	0.23	0.075
C1-C2	π	C5-C6	π*	19.26	0.27	0.063
C1-C6	∂	C5-C6	∂*	3.77	1.27	0.062
C1-C6	∂	N23-O24	∂*	2.31	1.14	0.042
C1-H7	∂	C2-C3	∂*	2.55	1.09	0.056
C1-H7	∂	C5-C6	∂*	4.44	1.08	0.062
C2-C3	∂	C3-C9	∂*	4.46	1.27	0.067
C2-C3	∂	C3-C10	∂*	5.30	1.39	0.077
C2-C3	∂	C4-N32	∂*	4.05	0.39	0.058
C2-C3	∂	C10-C11	∂*	4.44	1.60	0.076
C3-C4	∂	C3-C10	∂*	5.75	1.41	0.081
C3-C4	π	C1-C2	π*	14.03	0.29	0.059
C3-C4	π	C5-C6	π*	23.80	0.28	0.074
C3-C4	π	N32-O34	π*	24.20	0.17	0.060
C3-C10	∂	C10-C11	∂*	12.73	1.66	0.130
C5-C6	π	C1-C2	π*	21.97	0.29	0.073
C5-C6	π	C3-C4	π*	14.60	0.29	0.060
C5-C6	π	N23-O24	π*	24.65	0.16	0.060
C10-C11	∂	C3-C10	∂*	12.38	1.49	0.121
C10-C11	∂	C11-C12	∂*	7.80	1.46	0.096
C10-C11	π	C12-C13	π*	13.63	0.43	0.068
C11-C12	∂	C10-C11	∂*	10.08	1.64	0.113
C11-C12	∂	C12-C13	∂*	16.12	1.81	0.152
C12-C13	∂	C11-C12	∂*	12.21	1.51	0.121
C12-C13	π	C10-C11	π*	12.77	0.40	0.064
C13-C14	∂	C12-C13	∂*	11.69	1.74	0.127
C14-C15	π	C16-C19	π*	19.27	0.28	0.066
C14-C15	π	C17-C21	π*	19.02	0.29	0.067
C16-C19	π	C14-C15	π*	18.55	0.28	0.066
C16-C19	π	C17-C21	π*	20.74	0.28	0.070
C17-C21	π	C14-C15	π*	21.05	0.28	0.069
C17-C21	π	C16-C19	π*	19.25	0.28	0.069
O33	Lp(2)	C4-N32	∂*	15.97	0.56	0.084
O33	Lp(2)	N32-O34	∂*	20.45	0.75	0.112
O33	Lp(3)	N32-O34	π*	181.53	0.14	0.148
O34	Lp(2)	C4-N32	∂*	16.56	0.56	0.086
O34	Lp(2)	N32-O33	∂*	21.01	0.75	0.114
C5-C6	π	C1-C2	π*	224.30	0.01	0.074
C10-C11	π	C12-C13	π*	29.46	0.04	0.046
N23-O24	π	C5-C6	π*	19.22	0.12	0.062
N32-O34	π	C3-C4	π*	22.17	0.13	0.064

Table S12. Natural bond orbital (NBO) analysis of **4** using B3LYP/6-31G (d,p).

Donor (i)	Type	Acceptor (j)	Type	E(2)a (kcal/mol)	E(j)_E(i)b (a.u.)	F(i,j)c (a.u.)
C1-C2	∂	C1-C6	∂^*	2.59	1.25	0.051
C1-C2	∂	C1-H7	∂^*	1.26	1.20	0.035
C1-C2	∂	C2-C3	∂^*	2.46	1.26	0.050
C1-C2	∂	C3-C9	∂^*	3.26	1.30	0.058
C1-C2	∂	C6-N21	∂^*	4.51	0.99	0.061
C_1-C_2	π	C_3-C_4	π^*	22.08	0.29	0.072
C_1-C_2	π	C_5-C_6	π^*	18.99	0.27	0.065
C1-C6	∂	C1-C2	∂^*	2.06	1.27	0.046
C1-C6	∂	C5-C6	∂^*	3.89	1.26	0.063
C1-C6	∂	N21-O22	∂^*	2.32	1.14	0.046
C1-H7	∂	C2-C3	∂^*	3.52	1.10	0.056
C1-H7	∂	C5-C6	∂^*	4.26	1.08	0.060
C1-H7	∂	C6-N21	∂^*	0.60	0.83	0.020
C_3-C_4	π	C_1-C_2	π^*	17.77	0.28	0.065
C_3-C_4	π	C_5-C_6	π^*	22.92	0.27	0.071
C_3-C_9	σ	C_9-C_{10}	σ^*	11.16	1.64	0.121
C_5-C_6	π	C_1-C_2	π^*	20.86	0.29	0.071
C_5-C_6	π	C_3-C_4	π^*	16.34	0.29	0.062
C_5-C_6	π	N_{21}-O_{23}	π^*	28.49	0.15	0.063
C_9-C_{10}	σ	C_3-C_9	σ^*	9.83	1.43	0.106
C_9-C_{10}	σ	C_{10}-C_{11}	σ^*	8.42	1.46	0.099
C_9-C_{10}	π	C_{11}-C_{12}	π^*	7.94	0.43	0.052
C_{10}-H_{11}	σ	C_9-C_{10}	σ^*	10.74	1.66	0.119
C_{10}-C_{11}	σ	C_{11}-C_{12}	σ^*	16.01	1.81	0.152
C_{11}-C_{12}	σ	C_{10}-C_{11}	σ^*	12.02	1.50	0.120
C_{11}-C_{12}	σ	C_{12}-C_{13}	σ	9.50	1.42	0.104
C_{11}-H_{12}	π	C_9-C_{10}	π^*	8.19	0.40	0.051
C_{12}-C_{13}	σ	C_{11}-C_{12}	σ^*	12.94	1.75	0.135
C_{13}-C_{14}	π	C_{15}-C_{18}	π^*	19.43	0.28	0.066
C_{13}-C_{14}	π	C_{16}-C_{20}	π^*	20.45	0.28	0.069
C_{15}-C_{18}	π	C_{13}-C_{14}	π^*	19.37	0.28	0.067
C_{15}-C_{18}	π	C_{16}-C_{20}	π^*	20.99	0.28	0.070
C_{16}-C_{20}	π	C_{13}-C_{14}	π^*	21.04	0.28	0.069
C_{16}-C_{20}	π	C_{15}-C_{18}	π^*	20.73	0.28	0.069
O22	LP(2)	C_6-N_{21}	σ^*	13.04	0.57	0.077
O22	LP(2)	N_{21}-O_{23}	σ^*	18.04	0.69	0.101
N27	LP(1)	C13-C14	σ^*	0.54	0.86	0.019
O_{22}	LP(2)	N_{21}-O_{23}	π^*	160.28	0.14	0.135
O_{23}	LP(2)	C_6-C_{21}	σ^*	12.97	0.57	0.077
O_{23}	LP(2)	N_{21}-O_{22}	σ^*	18.10	0.69	0.101
N_{24}	LP(1)	C_{18}-C_{20}	σ^*	5.95	0.87	0.065
N_{27}	LP(1)	C_{14}-C_{16}	σ^*	5.63	0.87	0.063
N_{30}	LP(1)	C_{18}-C_{20}	σ^*	8.01	0.88	0.075
C_3-C_4	π	C_9-C_{10}	π^*	27.82	0.09	0.088

Table S12. *Cont.*

C_5-C_6	π	C_3-C_4	π^*	224.17	0.01	0.083
C_9-C_{10}	π	C_{11}-C_{12}	π^*	20.40	0.04	0.081
C_9-C_{10}	π	C_{11}-C_{12}	π^*	18.10	0.04	0.081
C_{13}-C_{14}	π	C_{11}-C_{12}	π^*	19.18	0.12	0.081
N_{21}-O_{23}	π	C_5-C_6	π^*	18.10	0.13	0.061

Table S13. Natural bond orbital (NBO) analysis of **5** using B3LYP/6-31G (d,p).

Donor (i)	Type	Acceptor (j)	Type	E(2)a (kcal/mol)	E(j)_E(i)b (a.u.)	F(i,j)c (a.u.)
C_1-C_2	π	C_3-C_4	π*	25.74	0.29	0.079
C_1-C_2	π	C_5-C_6	π*	14.51	0.28	0.057
C_1-C_2	π	N_{33}-O_{34}	π*	25.25	0.17	0.063
C_3-C_4	σ	C_3-C_9	σ*	5.02	1.35	0.074
C_3-C_4	π	C_1-C_2	π*	14	0.29	0.058
C_3-C_4	π	C_5-C_6	π*	25.07	0.29	0.077
C_3-C_4	π	N_{30}-O_{32}	π*	23.86	0.17	0.061
C_3-C_9	σ	C_9-C_{10}	σ*	11.9	1.67	0.126
C_5-C_6	π	C_1-C_2	π*	25.93	0.28	0.078
C_5-C_6	π	C_3-C_4	π*	14.12	0.29	0.058
C_5-C_6	π	N_{21}-O_{23}	π*	26.96	0.16	0.064
C_9-C_{10}	σ	C_3-C_9	σ*	10.84	1.42	0.111
C_9-C_{10}	σ	C_{10}-C_{11}	σ*	8.67	1.48	0.101
C_9-C_{10}	π	C_{11}-C_{12}	π*	15.38	0.42	0.072
C_{10}-C_{11}	σ	C_9-C_{10}	σ*	11.27	1.67	0.122
C_{10}-C_{11}	σ	C_{11}-C_{12}	σ*	15.22	1.78	0.147
C_{11}-C_{12}	σ	C_{10}-C_{11}	σ*	11.61	1.5	0.118
C_{11}-C_{12}	σ	C_{12}-C_{13}	σ*	8.8	1.42	0.1
C_{11}-C_{12}	π	C_9-C_{10}	π*	14.05	0.4	0.067
C_{12}-C_{13}	σ	C_{11}-C_{12}	σ*	11.8	1.73	0.127
C_{13}-C_{14}	π	C_{15}-C_{18}	π*	19.14	0.28	0.066
C_{13}-C_{14}	π	C_{16}-C_{20}	π*	19.96	0.29	0.068
C_{15}-C_{18}	π	C_{13}-C_{14}	π*	18.93	0.28	0.066
C_{15}-C_{18}	π	C_{16}-C_{20}	π*	21.07	0.28	0.07
C_{16}-C_{20}	π	C_{13}-C_{14}	π*	21.13	0.28	0.069
C_{16}-C_{20}	π	C_{15}-C_{18}	π*	20.36	0.28	0.069
N_{21}-O_{23}	π	N_{21}-O_{23}	π*	7.61	0.31	0.053
N_{30}-O_{32}	π	N_{30}-O_{32}	π*	7.52	0.32	0.053
N_{33}-O_{34}	π	N_{33}-O_{34}	π*	7.42	0.32	0.052
O_{22}	LP_2	C_6-N_{21}	σ*	13.8	0.55	0.078
O_{22}	LP_2	N_{21}-O_{23}	σ*	17.64	0.69	0.1
O_{22}	LP_3	N_{21}-O_{23}	π*	161.03	0.14	0.134

Table S13. *Cont.*

O_{23}	LP_2	C_6-N_{21}	σ*	13.83	0.55	0.078
O_{23}	LP_2	N_{21}-O_{22}	σ*	17.93	0.69	0.1
N_{24}	LP_1	C_{16}-C_{20}	π*	5.17	0.33	0.04
N_{24}	LP_1	C_{18}-C_{20}	σ*	5.83	0.87	0.064
N_{27}	LP_1	C_{14}-C_{16}	σ*	5.47	0.87	0.062
N_{27}	LP_1	C_{16}-C_{20}	π*	5.17	0.33	0.04
O_{31}	LP_2	C_4-N_{30}	σ*	14.85	0.56	0.082
O_{31}	LP_2	N_{30}-O_{32}	σ*	18.72	0.72	0.105
O_{31}	LP_3	N_{30}-O_{32}	π*	166.59	0.14	0.139
O_{32}	LP_2	C_4-N_{30}	σ*	14.38	0.56	0.081
O_{32}	LP_2	N_{30}-O_{31}	σ*	18.71	0.72	0.105
O_{34}	LP_2	C2-N_{33}	σ*	15.09	0.55	0.081
O_{34}	LP_2	N_{33}-O_{35}	σ*	18.23	0.71	0.103
O_{35}	LP_2	C_2-N_{33}	σ*	15.06	0.55	0.081
O_{35}	LP_2	N_{33}-O_{34}	σ*	18.41	0.71	0.103
O_{35}	LP_3	N_{33}-O_{34}	π*	164.68	0.14	0.138

Table S14. Natural bond orbital (NBO) analysis of **6** using B3LYP/6-31G (d,p).

Donor (i)	Type	Acceptor (j)	Type	E(2)a (kcal/mol)	E(j)_E(i)b (a.u.)	F(i,j)c (a.u.)
C2-H3	∂	O5-C6	∂*	0.52	0.94	0.020
C2-H3	∂	C5-C11	π*	4.17	0.56	0.044
C5-C11	π	O1-C6	π*	19.83	0.29	0.069
C5C11	π	C13-C21	π*	10.34	0.30	0.052
C7-C24	π	O1-C6	π*	19.24	0.30	0.068
C7-C24	π	C26-C34	π*	10.35	0.30	0.052
C13-C21	π	C5-C11	π*	12.01	0.30	0.058
C13-C21	π	C14-C16	π*	18.28	0.28	0.066
C13-C21	π	C18-C19	π*	22.22	0.28	0.07
C14-C16	π	C13-C21	π*	20.13	0.29	0.068
C14-C16	π	C18-C19	π*	20.78	0.28	0.069
C16-C18	∂	C18-C19	∂*	5.06	1.27	0.067
C18-C19	π	C13-C21	π*	19.47	0.29	0.069
C18-C19	π	C14-C16	π*	19.96	0.29	0.057
C26-C34	π	C7-C24	π*	11.59	0.31	0.066
C26-C34	π	C27-C29	π*	18.14	0.29	0.071
C26-C34	π	C31-C32	π*	22.40	0.28	0.068
C27-C29	π	C26-C34	π*	20.36	0.28	0.069
C27-C29	π	C31-C32	π*	20.88	0.28	0.067
C31-C32	π	C26-C34	π*	19.38	0.29	0.069
C31-C32	π	C27-C29	π*	19.93	0.29	0.068
C37-C45	π	C38-C40	π*	19.46	0.30	0.069
C37-C45	π	C42-C43	π*	18.36	0.30	0.066
C38-C40	π	C37-C45	π*	20.35	0.27	0.067
C38-C40	π	C42-C43	π*	20.35	0.28	0.068
C42-C43	π	C37-C45	π*	21.44	0.28	0.069
C42-C43	π	C38-C40	π*	19.15	0.29	0.068

Table S14. *Cont.*

C42-C43	π	C57-N58	π^*	11.05	0.38	0.063
O1	LP(2)	C5-C6	∂^*	19.09	0.69	0.104
O1	LP(2)	C6-C7	∂^*	19.32	0.68	0.104
F47	LP(3)	C23-F48	∂^*	11.00	0.66	0.077
F47	LP(3)	C23-F49	∂^*	9.62	0.66	0.072
F48	LP(3)	C23-F47	∂^*	11.10	0.66	0.077
F48	LP(3)	C23-F49	∂^*	9.62	0.66	0.072
F49	LP(3)	C23-F47	∂^*	10.31	0.65	0.074
F49	LP(3)	C23-F48	∂^*	10.24	0.65	0.074
O51	LP(2)	C37-S53	∂^*	17.65	0.44	0.079
O51	LP(2)	N50-S53	∂^*	9.11	0.40	0.055
O51	LP(3)	N50-S53	∂^*	14.88	0.40	0.070
O51	LP(3)	O52-S53	∂^*	18.16	0.57	0.092
O52	LP(2)	C37-S53	∂^*	17.57	0.44	0.079
O52	LP(2)	N50-S53	∂^*	7.75	0.41	0.051
O52	LP(3)	N50-S53	∂^*	18.96	0.40	0.079
O52	LP(3)	O51-S53	∂^*	16.06	0.58	0.088
F54	LP(3)	C36-F55	∂^*	9.50	0.66	0.071
F54	LP(3)	C36-F56	∂^*	11.08	0.66	0.077
F55	LP(3)	C36-F54	∂^*	10.35	0.65	0.074
F55	LP(3)	C36-F56	∂^*	10.29	0.65	0.074
F56	LP(3)	C36-F54	∂^*	11.10	0.66	0.077
F56	LP(3)	C36-F55	∂^*	9.53	0.66	0.071
N58	LP(1)	C42-C57	∂^*	11.33	0.87	0.088

a) ED/e is the electron density of donor and acceptor of NBO orbitals.
b) E(2) means energy of hyper conjugative interaction (stabilization energy).
c) E(j)_E(i) is the energy difference between donor and acceptor i and j NBO orbitals.
d) F(i,j) is the Fock matrix element between i and j NBO orbitals.
